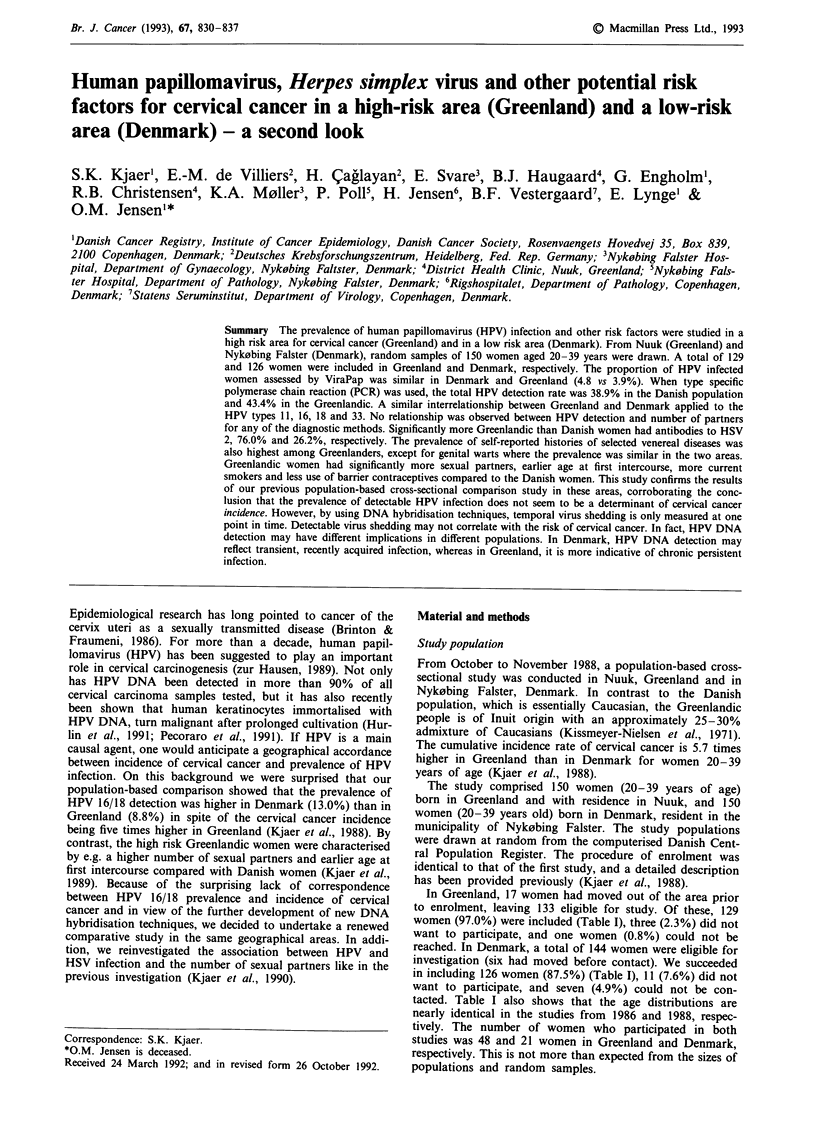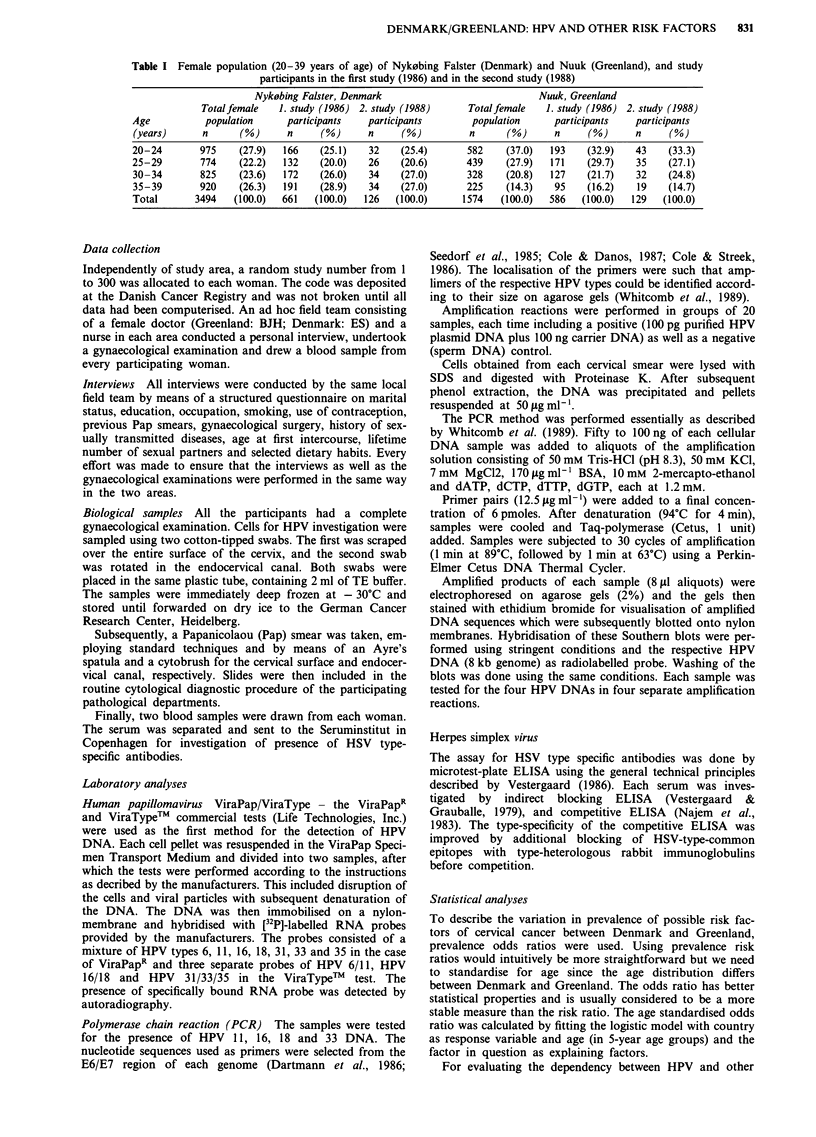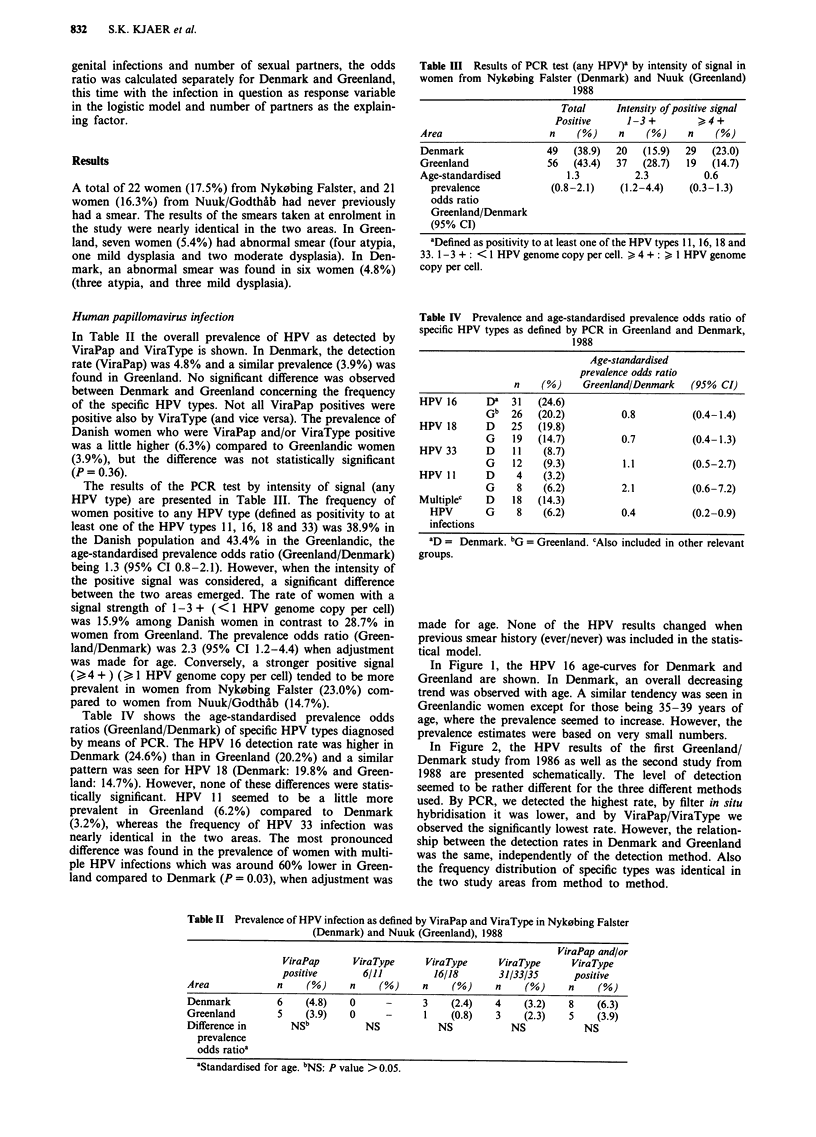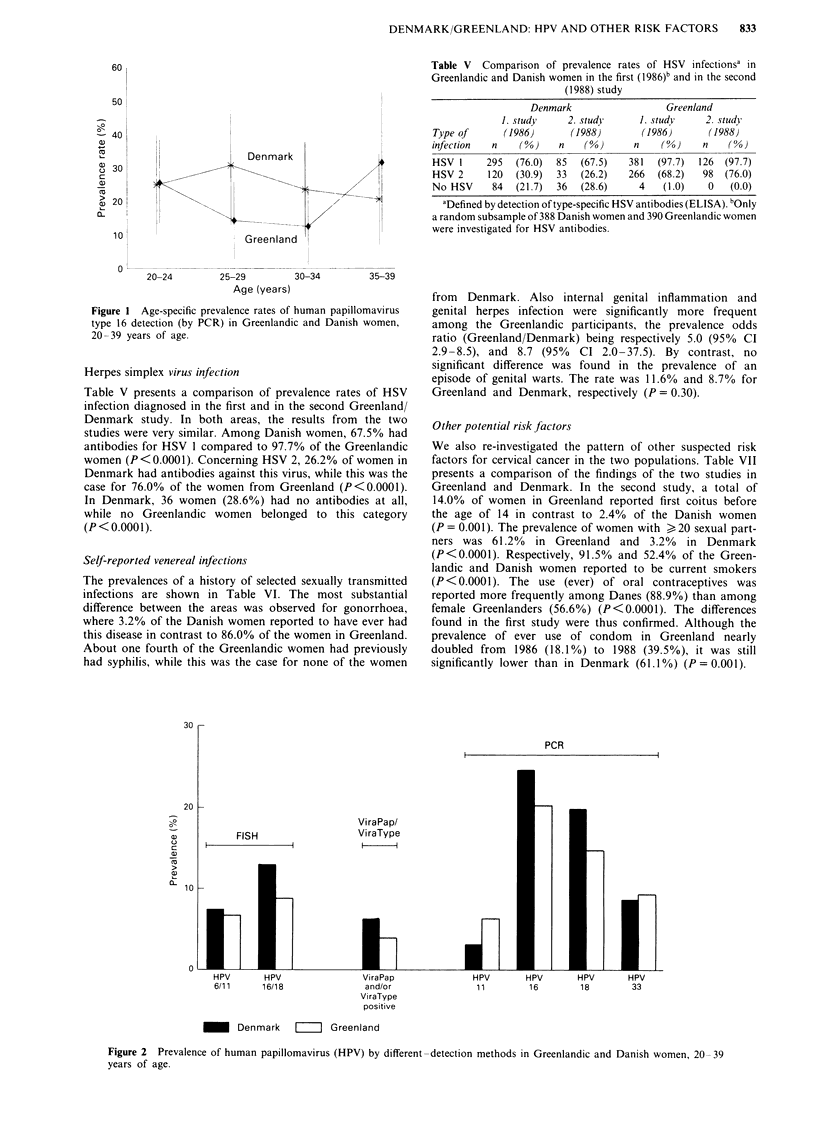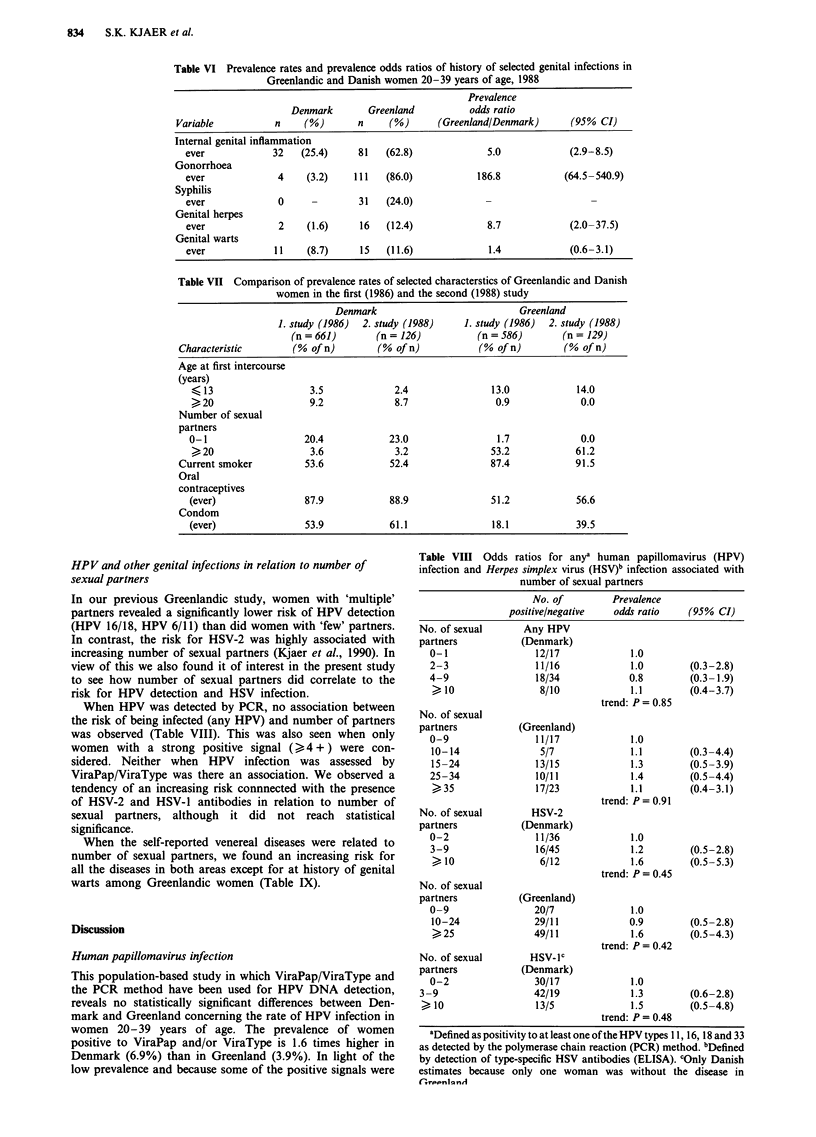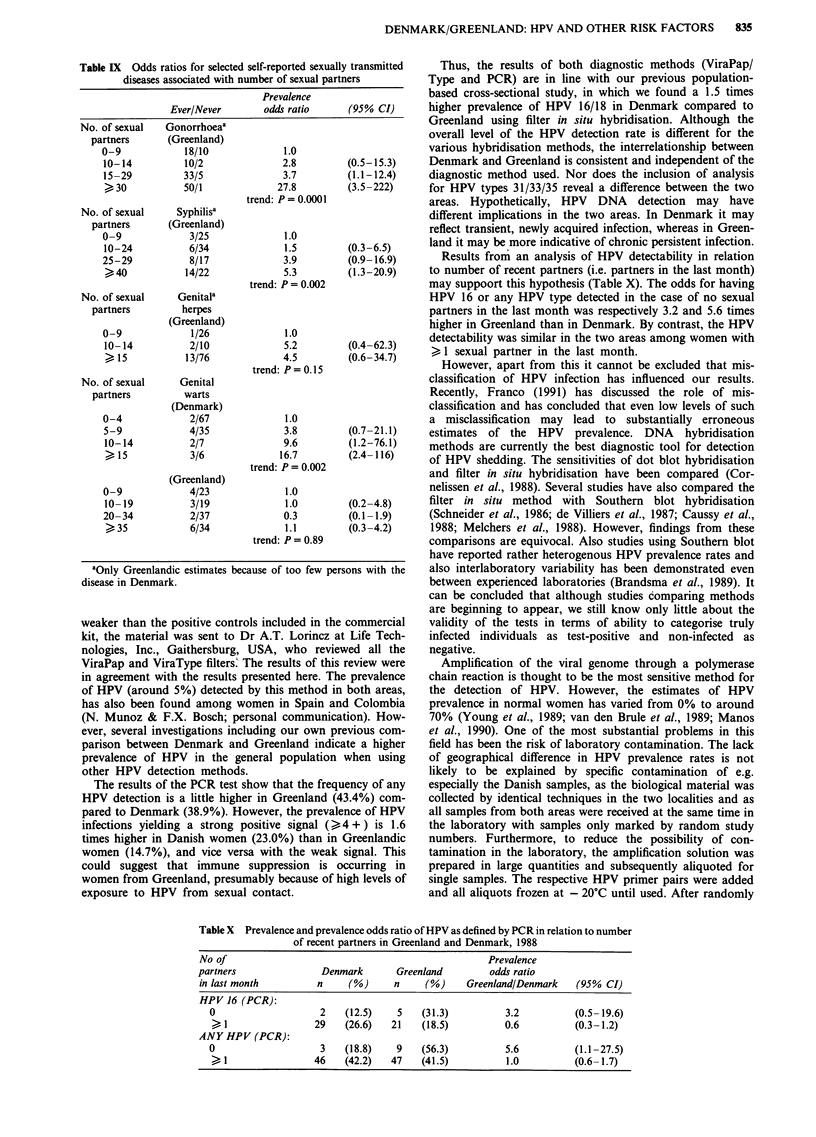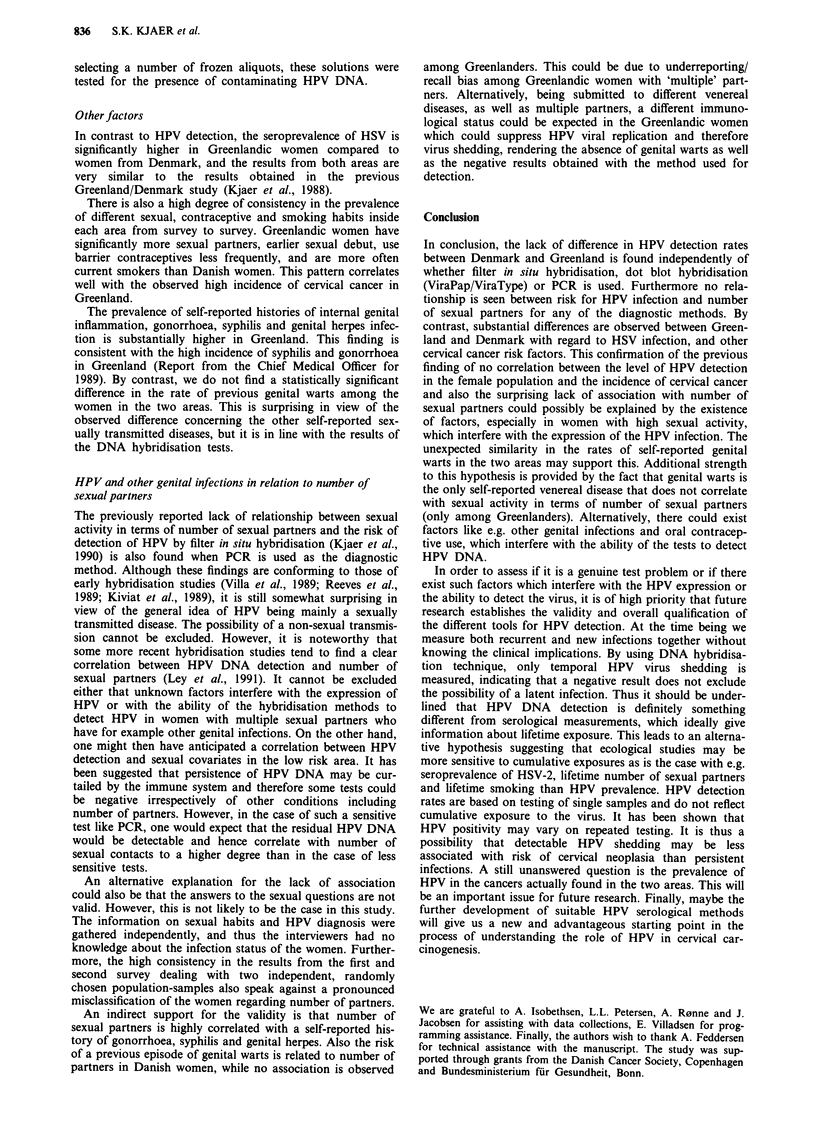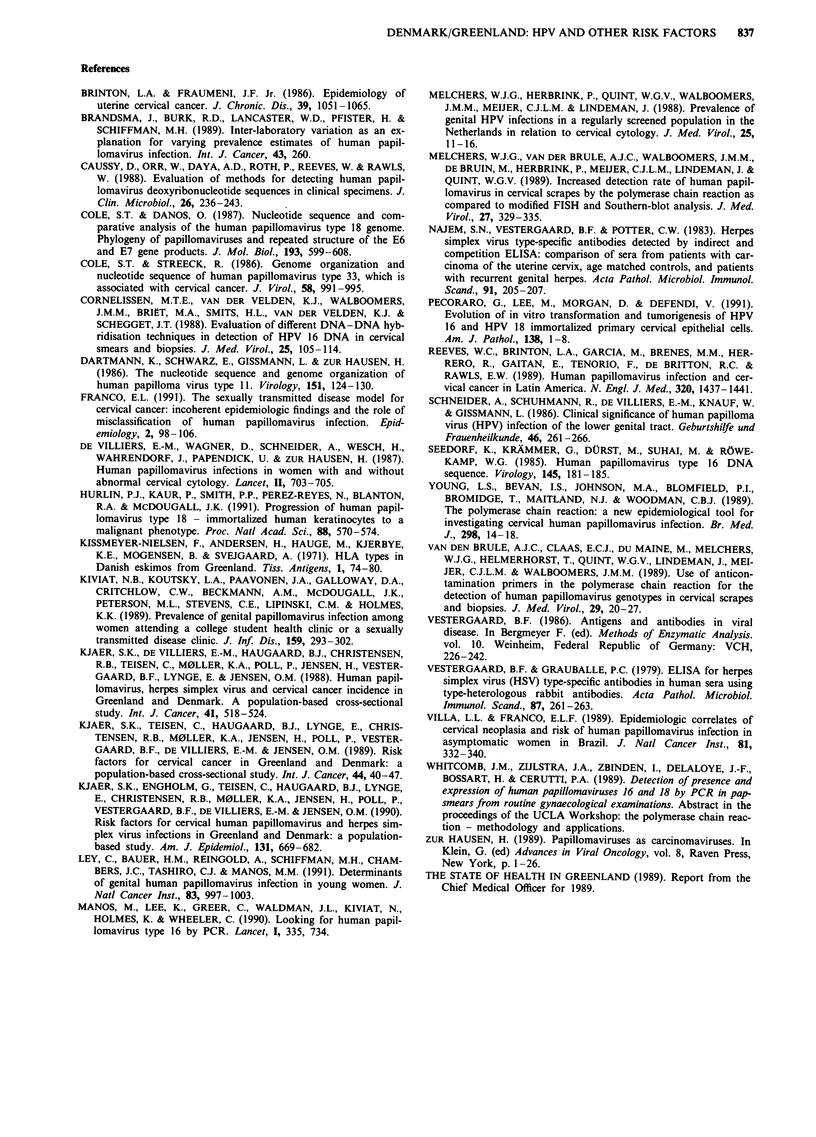# Human papillomavirus, herpes simplex virus and other potential risk factors for cervical cancer in a high-risk area (Greenland) and a low-risk area (Denmark)--a second look.

**DOI:** 10.1038/bjc.1993.152

**Published:** 1993-04

**Authors:** S. K. Kjaer, E. M. de Villiers, H. Cağlayan, E. Svare, B. J. Haugaard, G. Engholm, R. B. Christensen, K. A. Møller, P. Poll, H. Jensen

**Affiliations:** Danish Cancer Registry, Institute of Cancer Epidemiology, Copenhagen.

## Abstract

The prevalence of human papillomavirus (HPV) infection and other risk factors were studied in a high risk area for cervical cancer (Greenland) and in a low risk area (Denmark). From Nuuk (Greenland) and Nykøbing Falster (Denmark), random samples of 150 women aged 20-39 years were drawn. A total of 129 and 126 women were included in Greenland and Denmark, respectively. The proportion of HPV infected women assessed by ViraPap was similar in Denmark and Greenland (4.8 vs 3.9%). When type specific polymerase chain reaction (PCR) was used, the total HPV detection rate was 38.9% in the Danish population and 43.4% in the Greenlandic. A similar interrelationship between Greenland and Denmark applied to the HPV types 11, 16, 18 and 33. No relationship was observed between HPV detection and number of partners for any of the diagnostic methods. Significantly more Greenlandic than Danish women had antibodies to HSV 2, 76.0% and 26.2%, respectively. The prevalence of self-reported histories of selected venereal diseases was also highest among Greenlanders, except for genital warts where the prevalence was similar in the two areas. Greenlandic women had significantly more sexual partners, earlier age at first intercourse, more current smokers and less use of barrier contraceptives compared to the Danish women. This study confirms the results of our previous population-based cross-sectional comparison study in these areas, corroborating the conclusion that the prevalence of detectable HPV infection does not seem to be a determinant of cervical cancer incidence. However, by using DNA hybridisation techniques, temporal virus shedding is only measured at one point in time. Detectable virus shedding may not correlate with the risk of cervical cancer. In fact, HPV DNA detection may have different implications in different populations. In Denmark, HPV DNA detection may reflect transient, recently acquired infection, whereas in Greenland, it is more indicative of chronic persistent infection.


Br. J. Cancer (1993), 67, 830-837 © Macmillan Press Ltd., 1993

### Human papillomavirus, *Herpes simplex* virus and other potential risk factors for cervical cancer in a high-risk area (Greenland) and a low-risk area (Denmark) – a second look

S.K. Kjaer[1], E.-M. de Villiers[2], H. Çağlayan[2], E. Svare[3], B.J. Haugaard[4], G. Engholm[1], R.B. Christensen[4], K.A. Møller[3], P. Poll[5], H. Jensen[6], B.F. Vestergaard[7], E. Lynge[1] & O.M. Jensen[1]*

[1]*Danish Cancer Registry, Institute of Cancer Epidemiology, Danish Cancer Society, Rosenvaengets Hovedvej 35, Box 839, 2100 Copenhagen, Denmark;* [2]*Deutsches Krebsforschungszentrum, Heidelberg, Fed. Rep. Germany;* [3]*Nykøbing Falster Hospital, Department of Gynaecology, Nykøbing Faltster, Denmark;* [4]*District Health Clinic, Nuuk, Greenland;* [5]*Nykøbing Falster Hospital, Department of Pathology, Nykøbing Falster, Denmark;* [6]*Rigshospitalet, Department of Pathology, Copenhagen, Denmark;* [7]*Statens Seruminstitut, Department of Virology, Copenhagen, Denmark.*

**Summary** The prevalence of human papillomavirus (HPV) infection and other risk factors were studied in a high risk area for cervical cancer (Greenland) and in a low risk area (Denmark). From Nuuk (Greenland) and Nykøbing Falster (Denmark), random samples of 150 women aged 20–39 years were drawn. A total of 129 and 126 women were included in Greenland and Denmark, respectively. The proportion of HPV infected women assessed by ViraPap was similar in Denmark and Greenland (4.8 *vs* 3.9%). When type specific polymerase chain reaction (PCR) was used, the total HPV detection rate was 38.9% in the Danish population and 43.4% in the Greenlandic. A similar interrelationship between Greenland and Denmark applied to the HPV types 11, 16, 18 and 33. No relationship was observed between HPV detection and number of partners for any of the diagnostic methods. Significantly more Greenlandic than Danish women had antibodies to HSV 2, 76.0% and 26.2%, respectively. The prevalence of self-reported histories of selected venereal diseases was also highest among Greenlanders, except for genital warts where the prevalence was similar in the two areas. Greenlandic women had significantly more sexual partners, earlier age at first intercourse, more current smokers and less use of barrier contraceptives compared to the Danish women. This study confirms the results of our previous population-based cross-sectional comparison study in these areas, corroborating the conclusion that the prevalence of detectable HPV infection does not seem to be a determinant of cervical cancer *incidence*. However, by using DNA hybridisation techniques, temporal virus shedding is only measured at one point in time. Detectable virus shedding may not correlate with the risk of cervical cancer. In fact, HPV DNA detection may have different implications in different populations. In Denmark, HPV DNA detection may reflect transient, recently acquired infection, whereas in Greenland, it is more indicative of chronic persistent infection.

Epidemiological research has long pointed to cancer of the cervix uteri as a sexually transmitted disease (Brinton & Fraumeni, 1986). For more than a decade, human papillomavirus (HPV) has been suggested to play an important role in cervical carcinogenesis (zur Hausen, 1989). Not only has HPV DNA been detected in more than 90% of all cervical carcinoma samples tested, but it has also recently been shown that human keratinocytes immortalised with HPV DNA, turn malignant after prolonged cultivation (Hurlin *et al*., 1991; Pecoraro *et al*., 1991). If HPV is a main causal agent, one would anticipate a geographical accordance between incidence of cervical cancer and prevalence of HPV infection. On this background we were surprised that our population-based comparison showed that the prevalence of HPV 16/18 detection was higher in Denmark (13.0%) than in Greenland (8.8%) in spite of the cervical cancer incidence being five times higher in Greenland (Kjaer *et al*., 1988). By contrast, the high risk Greenlandic women were characterised by e.g. a higher number of sexual partners and earlier age at first intercourse compared with Danish women (Kjaer *et al*., 1989). Because of the surprising lack of correspondence between HPV 16/18 prevalence and incidence of cervical cancer and in view of the further development of new DNA hybridisation techniques, we decided to undertake a renewed comparative study in the same geographical areas. In addition, we reinvestigated the association between HPV and HSV infection and the number of sexual partners like in the previous investigation (Kjaer *et al*., 1990).

Correspondence: S.K. Kjaer.
*O.M. Jensen is deceased.
Received 24 March 1992; and in revised form 26 October 1992.

### Material and methods

### *Study population*

From October to November 1988, a population-based cross-sectional study was conducted in Nuuk, Greenland and in Nykøbing Falster, Denmark. In contrast to the Danish population, which is essentially Caucasian, the Greenlandic people is of Inuit origin with an approximately 25–30% admixture of Caucasians (Kissmeyer-Nielsen *et al*., 1971). The cumulative incidence rate of cervical cancer is 5.7 times higher in Greenland than in Denmark for women 20–39 years of age (Kjaer *et al*., 1988).

The study comprised 150 women (20–39 years of age) born in Greenland and with residence in Nuuk, and 150 women (20–39 years old) born in Denmark, resident in the municipality of Nykøbing Falster. The study populations were drawn at random from the computerised Danish Central Population Register. The procedure of enrolment was identical to that of the first study, and a detailed description has been provided previously (Kjaer *et al*., 1988).

In Greenland, 17 women had moved out of the area prior to enrolment, leaving 133 eligible for study. Of these, 129 women (97.0%) were included (Table I), three (2.3%) did not want to participate, and one women (0.8%) could not be reached. In Denmark, a total of 144 women were eligible for investigation (six had moved before contact). We succeeded in including 126 women (87.5%) (Table I), 11 (7.6%) did not want to participate, and seven (4.9%) could not be contacted. Table I also shows that the age distributions are nearly identical in the studies from 1986 and 1988, respectively. The number of women who participated in both studies was 48 and 21 women in Greenland and Denmark, respectively. This is not more than expected from the sizes of populations and random samples.

**Table I** Female population (20–39 years of age) of Nykøbing Falster (Denmark) and Nuuk (Greenland), and study participants in the first study (1986) and in the second study (1988)

| | *Nykøbing Falster, Denmark* | | | | | | *Nuuk, Greenland* | | | | | |
|---|---|---|---|---|---|---|---|---|---|---|---|---|
| | *Total female population* | | *1. study (1986) participants* | | *2. study (1988) participants* | | *Total female population* | | *1. study (1986) participants* | | *2. study (1988) participants* | |
| *Age (years)* | *n* | *(%)* | *n* | *(%)* | *n* | *(%)* | *n* | *(%)* | *n* | *(%)* | *n* | *(%)* |
| 20–24 | 975 | (27.9) | 166 | (25.1) | 32 | (25.4) | 582 | (37.0) | 193 | (32.9) | 43 | (33.3) |
| 25–29 | 774 | (22.2) | 132 | (20.0) | 26 | (20.6) | 439 | (27.9) | 171 | (29.7) | 35 | (27.1) |
| 30–34 | 825 | (23.6) | 172 | (26.0) | 34 | (27.0) | 328 | (20.8) | 127 | (21.7) | 32 | (24.8) |
| 35–39 | 920 | (26.3) | 191 | (28.9) | 34 | (27.0) | 225 | (14.3) | 95 | (16.2) | 19 | (14.7) |
| Total | 3494 | (100.0) | 661 | (100.0) | 126 | (100.0) | 1574 | (100.0) | 586 | (100.0) | 129 | (100.0) |

### *Data collection*

Independently of study area, a random study number from 1 to 300 was allocated to each woman. The code was deposited at the Danish Cancer Registry and was not broken until all data had been computerised. An ad hoc field team consisting of a female doctor (Greenland: BJH; Denmark: ES) and a nurse in each area conducted a personal interview, undertook a gynaecological examination and drew a blood sample from every participating woman.

*Interviews* All interviews were conducted by the same local field team by means of a structured questionnaire on marital status, education, occupation, smoking, use of contraception, previous Pap smears, gynaecological surgery, history of sexually transmitted diseases, age at first intercourse, lifetime number of sexual partners and selected dietary habits. Every effort was made to ensure that the interviews as well as the gynaecological examinations were performed in the same way in the two areas.

*Biological samples* All the participants had a complete gynaecological examination. Cells for HPV investigation were sampled using two cotton-tipped swabs. The first was scraped over the entire surface of the cervix, and the second swab was rotated in the endocervical canal. Both swabs were placed in the same plastic tube, containing 2 ml of TE buffer. The samples were immediately deep frozen at −30°C and stored until forwarded on dry ice to the German Cancer Research Center, Heidelberg.

Subsequently, a Papanicolaou (Pap) smear was taken, employing standard techniques and by means of an Ayre's spatula and a cytobrush for the cervical surface and endocervical canal, respectively. Slides were then included in the routine cytological diagnostic procedure of the participating pathological departments.

Finally, two blood samples were drawn from each woman. The serum was separated and sent to the Seruminstitut in Copenhagen for investigation of presence of HSV type-specific antibodies.

### *Laboratory analyses*

*Human papillomavirus* ViraPap/ViraType – the ViraPap^R^ and ViraType™ commercial tests (Life Technologies, Inc.) were used as the first method for the detection of HPV DNA. Each cell pellet was resuspended in the ViraPap Specimen Transport Medium and divided into two samples, after which the tests were performed according to the instructions as decribed by the manufacturers. This included disruption of the cells and viral particles with subsequent denaturation of the DNA. The DNA was then immobilised on a nylon-membrane and hybridised with [$^{32}$P]-labelled RNA probes provided by the manufacturers. The probes consisted of a mixture of HPV types 6, 11, 16, 18, 31, 33 and 35 in the case of ViraPap^R^ and three separate probes of HPV 6/11, HPV 16/18 and HPV 31/33/35 in the ViraType™ test. The presence of specifically bound RNA probe was detected by autoradiography.

*Polymerase chain reaction (PCR)* The samples were tested for the presence of HPV 11, 16, 18 and 33 DNA. The nucleotide sequences used as primers were selected from the E6/E7 region of each genome (Dartmann *et al.*, 1986; Seedorf *et al.*, 1985; Cole & Danos, 1987; Cole & Streek, 1986). The localisation of the primers were such that amplimers of the respective HPV types could be identified according to their size on agarose gels (Whitcomb *et al.*, 1989).

Amplification reactions were performed in groups of 20 samples, each time including a positive (100 pg purified HPV plasmid DNA plus 100 ng carrier DNA) as well as a negative (sperm DNA) control.

Cells obtained from each cervical smear were lysed with SDS and digested with Proteinase K. After subsequent phenol extraction, the DNA was precipitated and pellets resuspended at 50 $\mu$g ml$^{-1}$.

The PCR method was performed essentially as described by Whitcomb *et al.* (1989). Fifty to 100 ng of each cellular DNA sample was added to aliquots of the amplification solution consisting of 50 mM Tris-HCl (pH 8.3), 50 mM KCl, 7 mM MgCl2, 170 $\mu$g ml$^{-1}$ BSA, 10 mM 2-mercapto-ethanol and dATP, dCTP, dTTP, dGTP, each at 1.2 mM.

Primer pairs (12.5 $\mu$g ml$^{-1}$) were added to a final concentration of 6 pmoles. After denaturation (94°C for 4 min), samples were cooled and Taq-polymerase (Cetus, 1 unit) added. Samples were subjected to 30 cycles of amplification (1 min at 89°C, followed by 1 min at 63°C) using a Perkin-Elmer Cetus DNA Thermal Cycler.

Amplified products of each sample (8 $\mu$l aliquots) were electrophoresed on agarose gels (2%) and the gels then stained with ethidium bromide for visualisation of amplified DNA sequences which were subsequently blotted onto nylon membranes. Hybridisation of these Southern blots were performed using stringent conditions and the respective HPV DNA (8 kb genome) as radiolabelled probe. Washing of the blots was done using the same conditions. Each sample was tested for the four HPV DNAs in four separate amplification reactions.

### Herpes simplex *virus*

The assay for HSV type specific antibodies was done by microtest-plate ELISA using the general technical principles described by Vestergaard (1986). Each serum was investigated by indirect blocking ELISA (Vestergaard & Grauballe, 1979), and competitive ELISA (Najem *et al.*, 1983). The type-specificity of the competitive ELISA was improved by additional blocking of HSV-type-common epitopes with type-heterologous rabbit immunoglobulins before competition.

### *Statistical analyses*

To describe the variation in prevalence of possible risk factors of cervical cancer between Denmark and Greenland, prevalence odds ratios were used. Using prevalence risk ratios would intuitively be more straightforward but we need to standardise for age since the age distribution differs between Denmark and Greenland. The odds ratio has better statistical properties and is usually considered to be a more stable measure than the risk ratio. The age standardised odds ratio was calculated by fitting the logistic model with country as response variable and age (in 5-year age groups) and the factor in question as explaining factors.

For evaluating the dependency between HPV and other genital infections and number of sexual partners, the odds ratio was calculated separately for Denmark and Greenland, this time with the infection in question as response variable in the logistic model and number of partners as the explaining factor.

### Results

A total of 22 women (17.5%) from Nykøbing Falster, and 21 women (16.3%) from Nuuk/Godthåb had never previously had a smear. The results of the smears taken at enrolment in the study were nearly identical in the two areas. In Greenland, seven women (5.4%) had abnormal smear (four atypia, one mild dysplasia and two moderate dysplasia). In Denmark, an abnormal smear was found in six women (4.8%) (three atypia, and three mild dysplasia).

### *Human papillomavirus infection*

In Table II the overall prevalence of HPV as detected by ViraPap and ViraType is shown. In Denmark, the detection rate (ViraPap) was 4.8% and a similar prevalence (3.9%) was found in Greenland. No significant difference was observed between Denmark and Greenland concerning the frequency of the specific HPV types. Not all ViraPap positives were positive also by ViraType (and vice versa). The prevalence of Danish women who were ViraPap and/or ViraType positive was a little higher (6.3%) compared to Greenlandic women (3.9%), but the difference was not statistically significant ($P = 0.36$).

The results of the PCR test by intensity of signal (any HPV type) are presented in Table III. The frequency of women positive to any HPV type (defined as positivity to at least one of the HPV types 11, 16, 18 and 33) was 38.9% in the Danish population and 43.4% in the Greenlandic, the age-standardised prevalence odds ratio (Greenland/Denmark) being 1.3 (95% CI 0.8–2.1). However, when the intensity of the positive signal was considered, a significant difference between the two areas emerged. The rate of women with a signal strength of 1–3 + (<1 HPV genome copy per cell) was 15.9% among Danish women in contrast to 28.7% in women from Greenland. The prevalence odds ratio (Greenland/Denmark) was 2.3 (95% CI 1.2–4.4) when adjustment was made for age. Conversely, a stronger positive signal (≥4 + ) (≥1 HPV genome copy per cell) tended to be more prevalent in women from Nykøbing Falster (23.0%) compared to women from Nuuk/Godthåb (14.7%).

Table IV shows the age-standardised prevalence odds ratios (Greenland/Denmark) of specific HPV types diagnosed by means of PCR. The HPV 16 detection rate was higher in Denmark (24.6%) than in Greenland (20.2%) and a similar pattern was seen for HPV 18 (Denmark: 19.8% and Greenland: 14.7%). However, none of these differences were statistically significant. HPV 11 seemed to be a little more prevalent in Greenland (6.2%) compared to Denmark (3.2%), whereas the frequency of HPV 33 infection was nearly identical in the two areas. The most pronounced difference was found in the prevalence of women with multiple HPV infections which was around 60% lower in Greenland compared to Denmark ($P = 0.03$), when adjustment was made for age. None of the HPV results changed when previous smear history (ever/never) was included in the statistical model.

In Figure 1, the HPV 16 age-curves for Denmark and Greenland are shown. In Denmark, an overall decreasing trend was observed with age. A similar tendency was seen in Greenlandic women except for those being 35–39 years of age, where the prevalence seemed to increase. However, the prevalence estimates were based on very small numbers.

In Figure 2, the HPV results of the first Greenland/Denmark study from 1986 as well as the second study from 1988 are presented schematically. The level of detection seemed to be rather different for the three different methods used. By PCR, we detected the highest rate, by filter *in situ* hybridisation it was lower, and by ViraPap/ViraType we observed the significantly lowest rate. However, the relationship between the detection rates in Denmark and Greenland was the same, independently of the detection method. Also the frequency distribution of specific types was identical in the two study areas from method to method.

**Table III** Results of PCR test (any HPV)[a] by intensity of signal in women from Nykøbing Falster (Denmark) and Nuuk (Greenland) 1988

| Area | *Total Positive* *n* | *(%)* | *Intensity of positive signal* *1–3 +* *n* | *(%)* | *≥4 +* *n* | *(%)* |
|---|---|---|---|---|---|---|
| Denmark | 49 | (38.9) | 20 | (15.9) | 29 | (23.0) |
| Greenland | 56 | (43.4) | 37 | (28.7) | 19 | (14.7) |
| Age-standardised prevalence odds ratio Greenland/Denmark (95% CI) | 1.3 (0.8–2.1) | | 2.3 (1.2–4.4) | | 0.6 (0.3–1.3) | |

[a]Defined as positivity to at least one of the HPV types 11, 16, 18 and 33. 1–3 + : <1 HPV genome copy per cell. ≥4 + : ≥1 HPV genome copy per cell.

**Table IV** Prevalence and age-standardised prevalence odds ratio of specific HPV types as defined by PCR in Greenland and Denmark, 1988

| | | *n* | *(%)* | *Age-standardised prevalence odds ratio Greenland/Denmark* | *(95% CI)* |
|---|---|---|---|---|---|
| HPV 16 | D[a] | 31 | (24.6) | | |
| | G[b] | 26 | (20.2) | 0.8 | (0.4–1.4) |
| HPV 18 | D | 25 | (19.8) | | |
| | G | 19 | (14.7) | 0.7 | (0.4–1.3) |
| HPV 33 | D | 11 | (8.7) | | |
| | G | 12 | (9.3) | 1.1 | (0.5–2.7) |
| HPV 11 | D | 4 | (3.2) | | |
| | G | 8 | (6.2) | 2.1 | (0.6–7.2) |
| Multiple[c] HPV infections | D | 18 | (14.3) | | |
| | G | 8 | (6.2) | 0.4 | (0.2–0.9) |

[a]D = Denmark. [b]G = Greenland. [c]Also included in other relevant groups.

**Table II** Prevalence of HPV infection as defined by ViraPap and ViraType in Nykøbing Falster (Denmark) and Nuuk (Greenland), 1988

| Area | *ViraPap positive* *n* | *(%)* | *ViraType 6/11* *n* | *(%)* | *ViraType 16/18* *n* | *(%)* | *ViraType 31/33/35* *n* | *(%)* | *ViraPap and/or ViraType positive* *n* | *(%)* |
|---|---|---|---|---|---|---|---|---|---|---|
| Denmark | 6 | (4.8) | 0 | – | 3 | (2.4) | 4 | (3.2) | 8 | (6.3) |
| Greenland | 5 | (3.9) | 0 | – | 1 | (0.8) | 3 | (2.3) | 5 | (3.9) |
| Difference in prevalence odds ratio[a] | NS[b] | | NS | | NS | | NS | | NS | |

[a]Standardised for age. [b]NS: $P$ value >0.05.

**Figure 1** Age-specific prevalence rates of human papillomavirus type 16 detection (by PCR) in Greenlandic and Danish women, 20–39 years of age.

### Herpes simplex *virus infection*

Table V presents a comparison of prevalence rates of HSV infection diagnosed in the first and in the second Greenland/Denmark study. In both areas, the results from the two studies were very similar. Among Danish women, 67.5% had antibodies for HSV 1 compared to 97.7% of the Greenlandic women ($P<0.0001$). Concerning HSV 2, 26.2% of women in Denmark had antibodies against this virus, while this was the case for 76.0% of the women from Greenland ($P<0.0001$). In Denmark, 36 women (28.6%) had no antibodies at all, while no Greenlandic women belonged to this category ($P<0.0001$).

### *Self-reported venereal infections*

The prevalences of a history of selected sexually transmitted infections are shown in Table VI. The most substantial difference between the areas was observed for gonorrhoea, where 3.2% of the Danish women reported to have ever had this disease in contrast to 86.0% of the women in Greenland. About one fourth of the Greenlandic women had previously had syphilis, while this was the case for none of the women from Denmark. Also internal genital inflammation and genital herpes infection were significantly more frequent among the Greenlandic participants, the prevalence odds ratio (Greenland/Denmark) being respectively 5.0 (95% CI 2.9–8.5), and 8.7 (95% CI 2.0–37.5). By contrast, no significant difference was found in the prevalence of an episode of genital warts. The rate was 11.6% and 8.7% for Greenland and Denmark, respectively ($P=0.30$).

**Table V** Comparison of prevalence rates of HSV infections[a] in Greenlandic and Danish women in the first (1986)[b] and in the second (1988) study

| | *Denmark* | | | | *Greenland* | | | |
|---|---|---|---|---|---|---|---|---|
| | *1. study (1986)* | | *2. study (1988)* | | *1. study (1986)* | | *2. study (1988)* | |
| *Type of infection* | *n* | *(%)* | *n* | *(%)* | *n* | *(%)* | *n* | *(%)* |
| HSV 1 | 295 | (76.0) | 85 | (67.5) | 381 | (97.7) | 126 | (97.7) |
| HSV 2 | 120 | (30.9) | 33 | (26.2) | 266 | (68.2) | 98 | (76.0) |
| No HSV | 84 | (21.7) | 36 | (28.6) | 4 | (1.0) | 0 | (0.0) |

[a]Defined by detection of type-specific HSV antibodies (ELISA). [b]Only a random subsample of 388 Danish women and 390 Greenlandic women were investigated for HSV antibodies.

### *Other potential risk factors*

We also re-investigated the pattern of other suspected risk factors for cervical cancer in the two populations. Table VII presents a comparison of the findings of the two studies in Greenland and Denmark. In the second study, a total of 14.0% of women in Greenland reported first coitus before the age of 14 in contrast to 2.4% of the Danish women ($P=0.001$). The prevalence of women with $\geqslant 20$ sexual partners was 61.2% in Greenland and 3.2% in Denmark ($P<0.0001$). Respectively, 91.5% and 52.4% of the Greenlandic and Danish women reported to be current smokers ($P<0.0001$). The use (ever) of oral contraceptives was reported more frequently among Danes (88.9%) than among female Greenlanders (56.6%) ($P<0.0001$). The differences found in the first study were thus confirmed. Although the prevalence of ever use of condom in Greenland nearly doubled from 1986 (18.1%) to 1988 (39.5%), it was still significantly lower than in Denmark (61.1%) ($P=0.001$).

**Figure 2** Prevalence of human papillomavirus (HPV) by different-detection methods in Greenlandic and Danish women, 20–39 years of age.

**Table VI** Prevalence rates and prevalence odds ratios of history of selected genital infections in Greenlandic and Danish women 20–39 years of age, 1988

| Variable | Denmark n | Denmark (%) | Greenland n | Greenland (%) | Prevalence odds ratio (Greenland/Denmark) | (95% CI) |
|---|---|---|---|---|---|---|
| Internal genital inflammation | | | | | | |
| ever | 32 | (25.4) | 81 | (62.8) | 5.0 | (2.9–8.5) |
| Gonorrhoea | | | | | | |
| ever | 4 | (3.2) | 111 | (86.0) | 186.8 | (64.5–540.9) |
| Syphilis | | | | | | |
| ever | 0 | – | 31 | (24.0) | – | – |
| Genital herpes | | | | | | |
| ever | 2 | (1.6) | 16 | (12.4) | 8.7 | (2.0–37.5) |
| Genital warts | | | | | | |
| ever | 11 | (8.7) | 15 | (11.6) | 1.4 | (0.6–3.1) |

**Table VII** Comparison of prevalence rates of selected characterstics of Greenlandic and Danish women in the first (1986) and the second (1988) study

| Characteristic | Denmark 1. study (1986) (n = 661) (% of n) | Denmark 2. study (1988) (n = 126) (% of n) | Greenland 1. study (1986) (n = 586) (% of n) | Greenland 2. study (1988) (n = 129) (% of n) |
|---|---|---|---|---|
| Age at first intercourse (years) | | | | |
| ⩽13 | 3.5 | 2.4 | 13.0 | 14.0 |
| ⩾20 | 9.2 | 8.7 | 0.9 | 0.0 |
| Number of sexual partners | | | | |
| 0–1 | 20.4 | 23.0 | 1.7 | 0.0 |
| ⩾20 | 3.6 | 3.2 | 53.2 | 61.2 |
| Current smoker | 53.6 | 52.4 | 87.4 | 91.5 |
| Oral contraceptives | | | | |
| (ever) | 87.9 | 88.9 | 51.2 | 56.6 |
| Condom | | | | |
| (ever) | 53.9 | 61.1 | 18.1 | 39.5 |

*HPV and other genital infections in relation to number of sexual partners*

In our previous Greenlandic study, women with 'multiple' partners revealed a significantly lower risk of HPV detection (HPV 16/18, HPV 6/11) than did women with 'few' partners. In contrast, the risk for HSV-2 was highly associated with increasing number of sexual partners (Kjaer *et al.*, 1990). In view of this we also found it of interest in the present study to see how number of sexual partners did correlate to the risk for HPV detection and HSV infection.

When HPV was detected by PCR, no association between the risk of being infected (any HPV) and number of partners was observed (Table VIII). This was also seen when only women with a strong positive signal (⩾4+) were considered. Neither when HPV infection was assessed by ViraPap/ViraType was there an association. We observed a tendency of an increasing risk connnected with the presence of HSV-2 and HSV-1 antibodies in relation to number of sexual partners, although it did not reach statistical significance.

When the self-reported venereal diseases were related to number of sexual partners, we found an increasing risk for all the diseases in both areas except for at history of genital warts among Greenlandic women (Table IX).

**Table VIII** Odds ratios for any[a] human papillomavirus (HPV) infection and *Herpes simplex* virus (HSV)[b] infection associated with number of sexual partners

| | No. of positive/negative | Prevalence odds ratio | (95% CI) |
|---|---|---|---|
| No. of sexual partners | Any HPV (Denmark) | | |
| 0–1 | 12/17 | 1.0 | |
| 2–3 | 11/16 | 1.0 | (0.3–2.8) |
| 4–9 | 18/34 | 0.8 | (0.3–1.9) |
| ⩾10 | 8/10 | 1.1 | (0.4–3.7) |
| | | trend: $P = 0.85$ | |
| No. of sexual partners | (Greenland) | | |
| 0–9 | 11/17 | 1.0 | |
| 10–14 | 5/7 | 1.1 | (0.3–4.4) |
| 15–24 | 13/15 | 1.3 | (0.5–3.9) |
| 25–34 | 10/11 | 1.4 | (0.5–4.4) |
| ⩾35 | 17/23 | 1.1 | (0.4–3.1) |
| | | trend: $P = 0.91$ | |
| No. of sexual partners | HSV-2 (Denmark) | | |
| 0–2 | 11/36 | 1.0 | |
| 3–9 | 16/45 | 1.2 | (0.5–2.8) |
| ⩾10 | 6/12 | 1.6 | (0.5–5.3) |
| | | trend: $P = 0.45$ | |
| No. of sexual partners | (Greenland) | | |
| 0–9 | 20/7 | 1.0 | |
| 10–24 | 29/11 | 0.9 | (0.5–2.8) |
| ⩾25 | 49/11 | 1.6 | (0.5–4.3) |
| | | trend: $P = 0.42$ | |
| No. of sexual partners | HSV-1[c] (Denmark) | | |
| 0–2 | 30/17 | 1.0 | |
| 3–9 | 42/19 | 1.3 | (0.6–2.8) |
| ⩾10 | 13/5 | 1.5 | (0.5–4.8) |
| | | trend: $P = 0.48$ | |

[a]Defined as positivity to at least one of the HPV types 11, 16, 18 and 33 as detected by the polymerase chain reaction (PCR) method. [b]Defined by detection of type-specific HSV antibodies (ELISA). [c]Only Danish estimates because only one woman was without the disease in Greenland.

### Discussion

*Human papillomavirus infection*

This population-based study in which ViraPap/ViraType and the PCR method have been used for HPV DNA detection, reveals no statistically significant differences between Denmark and Greenland concerning the rate of HPV infection in women 20–39 years of age. The prevalence of women positive to ViraPap and/or ViraType is 1.6 times higher in Denmark (6.9%) than in Greenland (3.9%). In light of the low prevalence and because some of the positive signals were

**Table IX** Odds ratios for selected self-reported sexually transmitted diseases associated with number of sexual partners

| | *Ever/Never* | *Prevalence odds ratio* | *(95% CI)* |
|---|---|---|---|
| No. of sexual partners | Gonorrhoea[a] (Greenland) | | |
| 0–9 | 18/10 | 1.0 | |
| 10–14 | 10/2 | 2.8 | (0.5–15.3) |
| 15–29 | 33/5 | 3.7 | (1.1–12.4) |
| ⩾30 | 50/1 | 27.8 | (3.5–222) |
| | | trend: $P = 0.0001$ | |
| No. of sexual partners | Syphilis[a] (Greenland) | | |
| 0–9 | 3/25 | 1.0 | |
| 10–24 | 6/34 | 1.5 | (0.3–6.5) |
| 25–29 | 8/17 | 3.9 | (0.9–16.9) |
| ⩾40 | 14/22 | 5.3 | (1.3–20.9) |
| | | trend: $P = 0.002$ | |
| No. of sexual partners | Genital[a] herpes (Greenland) | | |
| 0–9 | 1/26 | 1.0 | |
| 10–14 | 2/10 | 5.2 | (0.4–62.3) |
| ⩾15 | 13/76 | 4.5 | (0.6–34.7) |
| | | trend: $P = 0.15$ | |
| No. of sexual partners | Genital warts (Denmark) | | |
| 0–4 | 2/67 | 1.0 | |
| 5–9 | 4/35 | 3.8 | (0.7–21.1) |
| 10–14 | 2/7 | 9.6 | (1.2–76.1) |
| ⩾15 | 3/6 | 16.7 | (2.4–116) |
| | | trend: $P = 0.002$ | |
| | (Greenland) | | |
| 0–9 | 4/23 | 1.0 | |
| 10–19 | 3/19 | 1.0 | (0.2–4.8) |
| 20–34 | 2/37 | 0.3 | (0.1–1.9) |
| ⩾35 | 6/34 | 1.1 | (0.3–4.2) |
| | | trend: $P = 0.89$ | |

[a]Only Greenlandic estimates because of too few persons with the disease in Denmark.

weaker than the positive controls included in the commercial kit, the material was sent to Dr A.T. Lorincz at Life Technologies, Inc., Gaithersburg, USA, who reviewed all the ViraPap and ViraType filters. The results of this review were in agreement with the results presented here. The prevalence of HPV (around 5%) detected by this method in both areas, has also been found among women in Spain and Colombia (N. Munoz & F.X. Bosch; personal communication). However, several investigations including our own previous comparison between Denmark and Greenland indicate a higher prevalence of HPV in the general population when using other HPV detection methods.

The results of the PCR test show that the frequency of any HPV detection is a little higher in Greenland (43.4%) compared to Denmark (38.9%). However, the prevalence of HPV infections yielding a strong positive signal (⩾4 +) is 1.6 times higher in Danish women (23.0%) than in Greenlandic women (14.7%), and vice versa with the weak signal. This could suggest that immune suppression is occurring in women from Greenland, presumably because of high levels of exposure to HPV from sexual contact.

Thus, the results of both diagnostic methods (ViraPap/Type and PCR) are in line with our previous population-based cross-sectional study, in which we found a 1.5 times higher prevalence of HPV 16/18 in Denmark compared to Greenland using filter *in situ* hybridisation. Although the overall level of the HPV detection rate is different for the various hybridisation methods, the interrelationship between Denmark and Greenland is consistent and independent of the diagnostic method used. Nor does the inclusion of analysis for HPV types 31/33/35 reveal a difference between the two areas. Hypothetically, HPV DNA detection may have different implications in the two areas. In Denmark it may reflect transient, newly acquired infection, whereas in Greenland it may be more indicative of chronic persistent infection.

Results from an analysis of HPV detectability in relation to number of recent partners (i.e. partners in the last month) may suppoort this hypothesis (Table X). The odds for having HPV 16 or any HPV type detected in the case of no sexual partners in the last month was respectively 3.2 and 5.6 times higher in Greenland than in Denmark. By contrast, the HPV detectability was similar in the two areas among women with ⩾1 sexual partner in the last month.

However, apart from this it cannot be excluded that misclassification of HPV infection has influenced our results. Recently, Franco (1991) has discussed the role of misclassification and has concluded that even low levels of such a misclassification may lead to substantially erroneous estimates of the HPV prevalence. DNA hybridisation methods are currently the best diagnostic tool for detection of HPV shedding. The sensitivities of dot blot hybridisation and filter *in situ* hybridisation have been compared (Cornelissen *et al.*, 1988). Several studies have also compared the filter *in situ* method with Southern blot hybridisation (Schneider *et al.*, 1986; de Villiers *et al.*, 1987; Caussy *et al.*, 1988; Melchers *et al.*, 1988). However, findings from these comparisons are equivocal. Also studies using Southern blot have reported rather heterogenous HPV prevalence rates and also interlaboratory variability has been demonstrated even between experienced laboratories (Brandsma *et al.*, 1989). It can be concluded that although studies comparing methods are beginning to appear, we still know only little about the validity of the tests in terms of ability to categorise truly infected individuals as test-positive and non-infected as negative.

Amplification of the viral genome through a polymerase chain reaction is thought to be the most sensitive method for the detection of HPV. However, the estimates of HPV prevalence in normal women has varied from 0% to around 70% (Young *et al.*, 1989; van den Brule *et al.*, 1989; Manos *et al.*, 1990). One of the most substantial problems in this field has been the risk of laboratory contamination. The lack of geographical difference in HPV prevalence rates is not likely to be explained by specific contamination of e.g. especially the Danish samples, as the biological material was collected by identical techniques in the two localities and as all samples from both areas were received at the same time in the laboratory with samples only marked by random study numbers. Furthermore, to reduce the possibility of contamination in the laboratory, the amplification solution was prepared in large quantities and subsequently aliquoted for single samples. The respective HPV primer pairs were added and all aliquots frozen at − 20°C until used. After randomly

**Table X** Prevalence and prevalence odds ratio of HPV as defined by PCR in relation to number of recent partners in Greenland and Denmark, 1988

| *No of partners in last month* | *Denmark n* | *(%)* | *Greenland n* | *(%)* | *Prevalence odds ratio Greenland/Denmark* | *(95% CI)* |
|---|---|---|---|---|---|---|
| *HPV 16 (PCR)*: | | | | | | |
| 0 | 2 | (12.5) | 5 | (31.3) | 3.2 | (0.5–19.6) |
| ⩾1 | 29 | (26.6) | 21 | (18.5) | 0.6 | (0.3–1.2) |
| *ANY HPV (PCR)*: | | | | | | |
| 0 | 3 | (18.8) | 9 | (56.3) | 5.6 | (1.1–27.5) |
| ⩾1 | 46 | (42.2) | 47 | (41.5) | 1.0 | (0.6–1.7) |

selecting a number of frozen aliquots, these solutions were tested for the presence of contaminating HPV DNA.

*Other factors*

In contrast to HPV detection, the seroprevalence of HSV is significantly higher in Greenlandic women compared to women from Denmark, and the results from both areas are very similar to the results obtained in the previous Greenland/Denmark study (Kjaer *et al.*, 1988).

There is also a high degree of consistency in the prevalence of different sexual, contraceptive and smoking habits inside each area from survey to survey. Greenlandic women have significantly more sexual partners, earlier sexual debut, use barrier contraceptives less frequently, and are more often current smokers than Danish women. This pattern correlates well with the observed high incidence of cervical cancer in Greenland.

The prevalence of self-reported histories of internal genital inflammation, gonorrhoea, syphilis and genital herpes infection is substantially higher in Greenland. This finding is consistent with the high incidence of syphilis and gonorrhoea in Greenland (Report from the Chief Medical Officer for 1989). By contrast, we do not find a statistically significant difference in the rate of previous genital warts among the women in the two areas. This is surprising in view of the observed difference concerning the other self-reported sexually transmitted diseases, but it is in line with the results of the DNA hybridisation tests.

*HPV and other genital infections in relation to number of sexual partners*

The previously reported lack of relationship between sexual activity in terms of number of sexual partners and the risk of detection of HPV by filter *in situ* hybridisation (Kjaer *et al.*, 1990) is also found when PCR is used as the diagnostic method. Although these findings are conforming to those of early hybridisation studies (Villa *et al.*, 1989; Reeves *et al.*, 1989; Kiviat *et al.*, 1989), it is still somewhat surprising in view of the general idea of HPV being mainly a sexually transmitted disease. The possibility of a non-sexual transmission cannot be excluded. However, it is noteworthy that some more recent hybridisation studies tend to find a clear correlation between HPV DNA detection and number of sexual partners (Ley *et al.*, 1991). It cannot be excluded either that unknown factors interfere with the expression of HPV or with the ability of the hybridisation methods to detect HPV in women with multiple sexual partners who have for example other genital infections. On the other hand, one might then have anticipated a correlation between HPV detection and sexual covariates in the low risk area. It has been suggested that persistence of HPV DNA may be curtailed by the immune system and therefore some tests could be negative irrespectively of other conditions including number of partners. However, in the case of such a sensitive test like PCR, one would expect that the residual HPV DNA would be detectable and hence correlate with number of sexual contacts to a higher degree than in the case of less sensitive tests.

An alternative explanation for the lack of association could also be that the answers to the sexual questions are not valid. However, this is not likely to be the case in this study. The information on sexual habits and HPV diagnosis were gathered independently, and thus the interviewers had no knowledge about the infection status of the women. Furthermore, the high consistency in the results from the first and second survey dealing with two independent, randomly chosen population-samples also speak against a pronounced misclassification of the women regarding number of partners.

An indirect support for the validity is that number of sexual partners is highly correlated with a self-reported history of gonorrhoea, syphilis and genital herpes. Also the risk of a previous episode of genital warts is related to number of partners in Danish women, while no association is observed among Greenlanders. This could be due to underreporting/recall bias among Greenlandic women with 'multiple' partners. Alternatively, being submitted to different venereal diseases, as well as multiple partners, a different immunological status could be expected in the Greenlandic women which could suppress HPV viral replication and therefore virus shedding, rendering the absence of genital warts as well as the negative results obtained with the method used for detection.

### Conclusion

In conclusion, the lack of difference in HPV detection rates between Denmark and Greenland is found independently of whether filter *in situ* hybridisation, dot blot hybridisation (ViraPap/ViraType) or PCR is used. Furthermore no relationship is seen between risk for HPV infection and number of sexual partners for any of the diagnostic methods. By contrast, substantial differences are observed between Greenland and Denmark with regard to HSV infection, and other cervical cancer risk factors. This confirmation of the previous finding of no correlation between the level of HPV detection in the female population and the incidence of cervical cancer and also the surprising lack of association with number of sexual partners could possibly be explained by the existence of factors, especially in women with high sexual activity, which interfere with the expression of the HPV infection. The unexpected similarity in the rates of self-reported genital warts in the two areas may support this. Additional strength to this hypothesis is provided by the fact that genital warts is the only self-reported venereal disease that does not correlate with sexual activity in terms of number of sexual partners (only among Greenlanders). Alternatively, there could exist factors like e.g. other genital infections and oral contraceptive use, which interfere with the ability of the tests to detect HPV DNA.

In order to assess if it is a genuine test problem or if there exist such factors which interfere with the HPV expression or the ability to detect the virus, it is of high priority that future research establishes the validity and overall qualification of the different tools for HPV detection. At the time being we measure both recurrent and new infections together without knowing the clinical implications. By using DNA hybridisation technique, only temporal HPV virus shedding is measured, indicating that a negative result does not exclude the possibility of a latent infection. Thus it should be underlined that HPV DNA detection is definitely something different from serological measurements, which ideally give information about lifetime exposure. This leads to an alternative hypothesis suggesting that ecological studies may be more sensitive to cumulative exposures as is the case with e.g. seroprevalence of HSV-2, lifetime number of sexual partners and lifetime smoking than HPV prevalence. HPV detection rates are based on testing of single samples and do not reflect cumulative exposure to the virus. It has been shown that HPV positivity may vary on repeated testing. It is thus a possibility that detectable HPV shedding may be less associated with risk of cervical neoplasia than persistent infections. A still unanswered question is the prevalence of HPV in the cancers actually found in the two areas. This will be an important issue for future research. Finally, maybe the further development of suitable HPV serological methods will give us a new and advantageous starting point in the process of understanding the role of HPV in cervical carcinogenesis.

We are grateful to A. Isobethsen, L.L. Petersen, A. Rønne and J. Jacobsen for assisting with data collections, E. Villadsen for programming assistance. Finally, the authors wish to thank A. Feddersen for technical assistance with the manuscript. The study was supported through grants from the Danish Cancer Society, Copenhagen and Bundesministerium für Gesundheit, Bonn.

### References

BRINTON, L.A. & FRAUMENI, J.F. Jr. (1986). Epidemiology of uterine cervical cancer. *J. Chronic. Dis.*, **39**, 1051–1065.

BRANDSMA, J., BURK, R.D., LANCASTER, W.D., PFISTER, H. & SCHIFFMAN, M.H. (1989). Inter-laboratory variation as an explanation for varying prevalence estimates of human papillomavirus infection. *Int. J. Cancer*, **43**, 260.

CAUSSY, D., ORR, W., DAYA, A.D., ROTH, P., REEVES, W. & RAWLS, W. (1988). Evaluation of methods for detecting human papillomavirus deoxyribonucleotide sequences in clinical specimens. *J. Clin. Microbiol.*, **26**, 236–243.

COLE, S.T. & DANOS, O. (1987). Nucleotide sequence and comparative analysis of the human papillomavirus type 18 genome. Phylogeny of papillomaviruses and repeated structure of the E6 and E7 gene products. *J. Mol. Biol.*, **193**, 599–608.

COLE, S.T. & STREECK, R. (1986). Genome organization and nucleotide sequence of human papillomavirus type 33, which is associated with cervical cancer. *J. Virol.*, **58**, 991–995.

CORNELISSEN, M.T.E., VAN DER VELDEN, K.J., WALBOOMERS, J.M.M., BRIËT, M.A., SMITS, H.L., VAN DER VELDEN, K.J. & SCHEGGET, J.T. (1988). Evaluation of different DNA–DNA hybridisation techniques in detection of HPV 16 DNA in cervical smears and biopsies. *J. Med. Virol.*, **25**, 105–114.

DARTMANN, K., SCHWARZ, E., GISSMANN, L. & ZUR HAUSEN, H. (1986). The nucleotide sequence and genome organization of human papilloma virus type 11. *Virology*, **151**, 124–130.

FRANCO, E.L. (1991). The sexually transmitted disease model for cervical cancer: incoherent epidemiologic findings and the role of misclassification of human papillomavirus infection. *Epidemiology*, **2**, 98–106.

DE VILLIERS, E.-M., WAGNER, D., SCHNEIDER, A., WESCH, H., WAHRENDORF, J., PAPENDICK, U. & ZUR HAUSEN, H. (1987). Human papillomavirus infections in women with and without abnormal cervical cytology. *Lancet*, **II**, 703–705.

HURLIN, P.J., KAUR, P., SMITH, P.P., PEREZ-REYES, N., BLANTON, R.A. & MCDOUGALL, J.K. (1991). Progression of human papillomavirus type 18 – immortalized human keratinocytes to a malignant phenotype. *Proc. Natl Acad. Sci.*, **88**, 570–574.

KISSMEYER-NIELSEN, F., ANDERSEN, H., HAUGE, M., KJERBYE, K.E., MOGENSEN, B. & SVEJGAARD, A. (1971). HLA types in Danish eskimos from Greenland. *Tiss. Antigens*, **1**, 74–80.

KIVIAT, N.B., KOUTSKY, L.A., PAAVONEN, J.A., GALLOWAY, D.A., CRITCHLOW, C.W., BECKMANN, A.M., MCDOUGALL, J.K., PETERSON, M.L., STEVENS, C.E., LIPINSKI, C.M. & HOLMES, K.K. (1989). Prevalence of genital papillomavirus infection among women attending a college student health clinic or a sexually transmitted disease clinic. *J. Inf. Dis.*, **159**, 293–302.

KJAER, S.K., DE VILLIERS, E.-M., HAUGAARD, B.J., CHRISTENSEN, R.B., TEISEN, C., MØLLER, K.A., POLL, P., JENSEN, H., VESTERGAARD, B.F., LYNGE, E. & JENSEN, O.M. (1988). Human papillomavirus, herpes simplex virus and cervical cancer incidence in Greenland and Denmark. A population-based cross-sectional study. *Int. J. Cancer*, **41**, 518–524.

KJAER, S.K., TEISEN, C., HAUGAARD, B.J., LYNGE, E., CHRISTENSEN, R.B., MØLLER, K.A., JENSEN, H., POLL, P., VESTERGAARD, B.F., DE VILLIERS, E.-M. & JENSEN, O.M. (1989). Risk factors for cervical cancer in Greenland and Denmark: a population-based cross-sectional study. *Int. J. Cancer*, **44**, 40–47.

KJAER, S.K., ENGHOLM, G., TEISEN, C., HAUGAARD, B.J., LYNGE, E., CHRISTENSEN, R.B., MØLLER, K.A., JENSEN, H., POLL, P., VESTERGAARD, B.F., DE VILLIERS, E.-M. & JENSEN, O.M. (1990). Risk factors for cervical human papillomavirus and herpes simplex virus infections in Greenland and Denmark: a population-based study. *Am. J. Epidemiol.*, **131**, 669–682.

LEY, C., BAUER, H.M., REINGOLD, A., SCHIFFMAN, M.H., CHAMBERS, J.C., TASHIRO, C.J. & MANOS, M.M. (1991). Determinants of genital human papillomavirus infection in young women. *J. Natl Cancer Inst.*, **83**, 997–1003.

MANOS, M., LEE, K., GREER, C., WALDMAN, J.L., KIVIAT, N., HOLMES, K. & WHEELER, C. (1990). Looking for human papillomavirus type 16 by PCR. *Lancet*, **I**, 335, 734.

MELCHERS, W.J.G., HERBRINK, P., QUINT, W.G.V., WALBOOMERS, J.M.M., MEIJER, C.J.L.M. & LINDEMAN, J. (1988). Prevalence of genital HPV infections in a regularly screened population in the Netherlands in relation to cervical cytology. *J. Med. Virol.*, **25**, 11–16.

MELCHERS, W.J.G., VAN DER BRULE, A.J.C., WALBOOMERS, J.M.M., DE BRUIN, M., HERBRINK, P., MEIJER, C.J.L.M., LINDEMAN, J. & QUINT, W.G.V. (1989). Increased detection rate of human papillomavirus in cervical scrapes by the polymerase chain reaction as compared to modified FISH and Southern-blot analysis. *J. Med. Virol.*, **27**, 329–335.

NAJEM, S.N., VESTERGAARD, B.F. & POTTER, C.W. (1983). Herpes simplex virus type-specific antibodies detected by indirect and competition ELISA: comparison of sera from patients with carcinoma of the uterine cervix, age matched controls, and patients with recurrent genital herpes. *Acta Pathol. Microbiol. Immunol. Scand.*, **91**, 205–207.

PECORARO, G., LEE, M., MORGAN, D. & DEFENDI, V. (1991). Evolution of in vitro transformation and tumorigenesis of HPV 16 and HPV 18 immortalized primary cervical epithelial cells. *Am. J. Pathol.*, **138**, 1–8.

REEVES, W.C., BRINTON, L.A., GARCIA, M., BRENES, M.M., HERRERO, R., GAITAN, E., TENORIO, F., DE BRITTON, R.C. & RAWLS, E.W. (1989). Human papillomavirus infection and cervical cancer in Latin America. *N. Engl. J. Med.*, **320**, 1437–1441.

SCHNEIDER, A., SCHUHMANN, R., DE VILLIERS, E.-M., KNAUF, W. & GISSMANN, L. (1986). Clinical significance of human papilloma virus (HPV) infection of the lower genital tract. *Geburtshilfe und Frauenheilkunde*, **46**, 261–266.

SEEDORF, K., KRÄMMER, G., DÜRST, M., SUHAI, M. & RÖWEKAMP, W.G. (1985). Human papillomavirus type 16 DNA sequence. *Virology*, **145**, 181–185.

YOUNG, L.S., BEVAN, I.S., JOHNSON, M.A., BLOMFIELD, P.I., BROMIDGE, T., MAITLAND, N.J. & WOODMAN, C.B.J. (1989). The polymerase chain reaction: a new epidemiological tool for investigating cervical human papillomavirus infection. *Br. Med. J.*, **298**, 14–18.

VAN DEN BRULE, A.J.C., CLAAS, E.C.J., DU MAINE, M., MELCHERS, W.J.G., HELMERHORST, T., QUINT, W.G.V., LINDEMAN, J., MEIJER, C.J.L.M. & WALBOOMERS, J.M.M. (1989). Use of anticontamination primers in the polymerase chain reaction for the detection of human papillomavirus genotypes in cervical scrapes and biopsies. *J. Med. Virol.*, **29**, 20–27.

VESTERGAARD, B.F. (1986). Antigens and antibodies in viral disease. In Bergmeyer F. (ed). *Methods of Enzymatic Analysis*. vol. 10. Weinheim, Federal Republic of Germany: VCH, 226–242.

VESTERGAARD, B.F. & GRAUBALLE, P.C. (1979). ELISA for herpes simplex virus (HSV) type-specific antibodies in human sera using type-heterologous rabbit antibodies. *Acta Pathol. Microbiol. Immunol. Scand.*, **87**, 261–263.

VILLA, L.L. & FRANCO, E.L.F. (1989). Epidemiologic correlates of cervical neoplasia and risk of human papillomavirus infection in asymptomatic women in Brazil. *J. Natl Cancer Inst.*, **81**, 332–340.

WHITCOMB, J.M., ZIJLSTRA, J.A., ZBINDEN, I., DELALOYE, J.-F., BOSSART, H. & CERUTTI, P.A. (1989). *Detection of presence and expression of human papillomaviruses 16 and 18 by PCR in papsmears from routine gynaecological examinations*. Abstract in the proceedings of the UCLA Workshop: the polymerase chain reaction – methodology and applications.

ZUR HAUSEN, H. (1989). Papillomaviruses as carcinomaviruses. In Klein, G. (ed) *Advances in Viral Oncology*, vol. 8, Raven Press, New York, p. 1–26.

THE STATE OF HEALTH IN GREENLAND (1989). Report from the Chief Medical Officer for 1989.